# Pre-Clinical and Clinical Applications of Small Interfering RNAs (siRNA) and Co-Delivery Systems for Pancreatic Cancer Therapy

**DOI:** 10.3390/cells10123348

**Published:** 2021-11-29

**Authors:** Sepideh Mirzaei, Mohammad Hossein Gholami, Hui Li Ang, Farid Hashemi, Ali Zarrabi, Amirhossein Zabolian, Kiavash Hushmandi, Masoud Delfi, Haroon Khan, Milad Ashrafizadeh, Gautam Sethi, Alan Prem Kumar

**Affiliations:** 1 Department of Biology, Faculty of Science, Islamic Azad University, Science and Research Branch, Tehran 1477893855, Iran; sepidehmirzaei.smv@gmail.com; 2 Faculty of Veterinary Medicine, Kazerun Branch, Islamic Azad University, Kazerun 7319866451, Iran; hoseingholami2020@yahoo.com; 3 Cancer Science Institute of Singapore and Department of Pharmacology, Yong Loo Lin School of Medicine, National University of Singapore, Singapore 117599, Singapore; e0336095@u.nus.edu; 4 Department of Comparative Biosciences, Faculty of Veterinary Medicine, University of Tehran, Tehran 1419963114, Iran; faridhashemi172@gmail.com; 5 Department of Biomedical Engineering, Faculty of Engineering and Natural Sciences, Istinye University, Sariyer, Istanbul 34396, Turkey; alizarrabi@sabanciuniv.edu; 6 Resident of Orthopedics, Department of Orthopedics, School of Medicine, 5th Azar Hospital, Golestan University of Medical Sciences, Golestan 4934174515, Iran; ah_zabolian@student.iautmu.ac.ir; 7 Department of Food Hygiene and Quality Control, Division of Epidemiology, Faculty of Veterinary Medicine, University of Tehran, Tehran 1419963114, Iran; houshmandi.kia7@ut.ac.ir; 8 Department of Chemical Sciences, Complesso Universitario Monte S. Angelo, University of Naples “Federico II”, 80126 Naples, Italy; masouddelfi51@gmail.com; 9 Department of Pharmacy, Abdul Wali Khan University, Mardan 23200, Pakistan; haroonkhan@awkum.edu.pk; 10 Faculty of Engineering and Natural Sciences, Sabanci University, Orta Mahalle, Üniversite Caddesi No. 27, Orhanlı, Tuzla, Istanbul 34956, Turkey; 11 Sabanci University Nanotechnology Research and Application Center (SUNUM), Tuzla, Istanbul 34956, Turkey; 12 Department of Pharmacology, Yong Loo Lin School of Medicine, National University of Singapore, Singapore 117600, Singapore; 13 NUS Centre for Cancer Research (N2CR), Yong Loo Lin School of Medicine, National University of Singapore, Singapore 119228, Singapore

**Keywords:** pancreatic cancer, small interfering RNA (siRNA), drug resistance, co-delivery, nanoparticles

## Abstract

Pancreatic cancer (PC) is one of the leading causes of death and is the fourth most malignant tumor in men. The epigenetic and genetic alterations appear to be responsible for development of PC. Small interfering RNA (siRNA) is a powerful genetic tool that can bind to its target and reduce expression level of a specific gene. The various critical genes involved in PC progression can be effectively targeted using diverse siRNAs. Moreover, siRNAs can enhance efficacy of chemotherapy and radiotherapy in inhibiting PC progression. However, siRNAs suffer from different off target effects and their degradation by enzymes in serum can diminish their potential in gene silencing. Loading siRNAs on nanoparticles can effectively protect them against degradation and can inhibit off target actions by facilitating targeted delivery. This can lead to enhanced efficacy of siRNAs in PC therapy. Moreover, different kinds of nanoparticles such as polymeric nanoparticles, lipid nanoparticles and metal nanostructures have been applied for optimal delivery of siRNAs that are discussed in this article. This review also reveals that how naked siRNAs and their delivery systems can be exploited in treatment of PC and as siRNAs are currently being applied in clinical trials, significant progress can be made by translating the current findings into the clinical settings.

## 1. Introduction

Despite advances in various treatment modalities, pancreatic cancer (PC) still remains an incurable disease. The incidence rate of PC has shown an increase and prognosis of patients is not favorable [1,2]. Based on new estimates, PC is the fourth most leading cause of cancer related death [3]. PC patients lack typical early symptoms and they are mainly diagnosed in advanced stages. In fact, there are no specific markers and symptoms for diagnosis of PC patients in early stages and that is why these patients are diagnosed in advanced stages, when cancer cells are not completely responsive to therapy. Although surgery is a potential strategy in PC therapy, as patients are generally diagnosed during local progression or distant metastasis, it is impossible to eradicate PC by surgery [4]. The 5-year survival rate for PC is less than 9%, therefore showing fatality of this cancer [5]. Despite many efforts in providing a novel and effective therapeutic for PC, survival rate of PC patients in last 40 years have not undergone significant improvement [6]. These statements demonstrate that scientists have not been successful in developing an efficient therapeutic against PC. One of the reasons for this failure is that there are a variety of signaling networks participating in cancer progression along with a few other oncogenic pathways that have not been identified yet [7,8,9,10,11,12,13]. Furthermore, currently applied therapies are not successful in eradicating cancer. For instance, anti-tumor compounds, often suffer from poor bioavailability [14,15]. This restricts their ability in suppressing cancer proliferation and metastasis. Moreover, genetic tools such as CRISPR/Cas9 and small interfering RNA (siRNA), despite their ability to facilitate gene silencing and inhibit cancer progression are unable to completely kill cancer cells [16,17,18,19]. This is due to presence of some limitations including their off-targeting feature, their degradation in blood circulation and other impediments such as blood-brain barrier (BBB) and blood-tumor barrier (BTB) [20,21,22,23,24]. Based on these facts, scientists should direct their efforts toward cancer therapeutics that are effective under both in vitro and in vivo conditions and then, such results can be translated into clinical settings for the treatment of cancer patients. To overcome aforementioned problems, there have been efforts in using methods for improving efficacy of drugs and genetic tools for cancer suppression. One of the most beneficial and well-known ways is using nanocarriers for cancer therapy [25]. In field of gene therapy, gene editing tools such as siRNA and CRISPR/Cas9 systems can be loaded on nanostructures to enhance their intracellular accumulation and prevent off-targeting feature and serum degradation [26,27,28,29,30]. Different nanoarchitectures can be applied for this purpose including carbon quantum dots [31], polymeric nanoparticles [32], lipid nanoparticles [33] and metal nanoparticles [34].

The present review focuses on the potential applications of siRNAs in treatment of PC based on pre-clinical studies. In vitro studies confirm the possible role of naked siRNA in suppressing PC progression via down-regulating tumor-promoting genes such as *NUF2*, *Survivin*, *RAP80*, *HIF-1α* and *hTERT*. However, efficacy of siRNA decreases markedly in vivo, but it is still capable of reducing gene expression of key genes. Based on the experiments, siRNAs can decrease growth and metastasis of PC cells and promote their sensitivity to therapy. Noteworthy, various kinds of nanostructures including micelles, liposomes, carbon-based nanomaterials and dendrimers among others have been used for delivery of siRNAs and in promoting their potential for gene down-regulation in PC treatment. These topics are discussed in the current review article. Furthermore, our review also emphasizes on the different molecular pathways that have been not extensively discussed in previous articles. Moreover, outline of our article is novel and is different from other reviews in this field [35,36,37,38,39,40,41].

## 2. SiRNAs: From Basic to Application in Cancer Therapy

**Figure 1:**
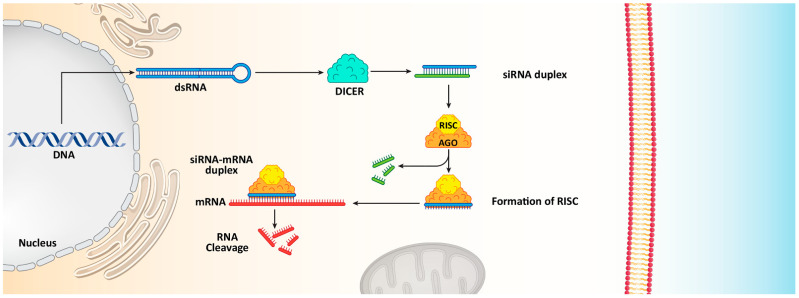
The functions of siRNA in reducing gene expression and causing mRNA cleavage.

siRNA as a key strategy of RNA interference (RNAi) is of importance in field of cancer therapy due to its potential in down-regulating expression of oncogenes [42]. siRNA molecules are double-stranded oligonucleotides with length of 20–25 base pairs capable of stimulating messenger RNA (mRNA) degradation and reducing gene expression [43]. The process of mRNA degradation is a little complex [44]. Although a variety of RNA degradation pathways have been identified, the basics of RNA degradation in bacteria, archaea and eukeryotic cells are similar [45]. Overall, there are three categories of enzymes capable of mediating RNA degradation including A) endonucelases with capacity of RNA degradation internally, 5′ exonucleases that can cut RNA from 5′ end to 3′ exonucleases that can cut RNA from 3′ end [46,47]. For the purpose of RNA degradation, siRNA is embedded to RNA-induced silencing complex (RISC) that also contains Argonaute protein (Ago-2) with ability of cleaving and eliminating passenger strand of siRNA duplex. RISC complex in accompany with single stranded guide RNA can recognize targeted mRNA via complementary base pairing [48,49]. Then, Ago-2 degrades mRNA complementary to antisense strand to provide nucleolytic cleavage to 5′ end of antisense siRNA strand (Figure 1) [50,51,52,53,54].

Currently, siRNAs can serve as powerful tool that scientists use in cancer therapy. Following identification of signaling networks responsible for cancer malignancy, siRNAs can be developed for targeting them. To date, a variety of studies have evaluated potentiality of siRNAs in gene silencing both in vitro and in vivo. For instance, Bcl-2 is an anti-apoptotic factor that its up-regulation is in favor of glioblastoma growth and viability. Using siRNA significantly diminishes Bcl-2 expression to induce apoptosis in glioblastoma cells, and promote their sensitivity to taxol chemotherapy [55]. Noteworthy, co-application of siRNA with anti-tumor compounds can exhibit synergistic anti-cancer effects. The CD73-siRNA is able to impair proliferation and progression of tumor cells that are of importance for enhancing doxorubicin sensitivity [56]. The Bcl-2-siRNA induces apoptotic cell death and reverses doxorubicin resistance in hepatocellular carcinoma [57]. The same strategy has been used for cisplatin sensitivity, such that using GPX4-siRNA and ABCC3-siRNA can induce drug sensitivity in glioblastoma and lung tumors, respectively [58,59]. Tumor-promoting factors such as poly (ADP-ribose) polymerase (PARP) and endothelial cell-specific molecule-1 (ESM-1) can be down-regulated by siRNA in suppressing proliferation and migration of cancer cells, respectively [60,61].

Low toxicity and high efficiency are potential benefits of siRNA [62,63]. However, siRNAs are associated with several limitations that should be addressed. Despite promising results in vitro [64], it has been found that siRNAs are not quite successful in causing gene silencing in vivo. More investigations have revealed that siRNAs could be degraded by RNase enzymes present in the serum [65]. They cannot sufficiently penetrate cell membrane, requiring nanocarriers for its delivery. Finally, their off-targeting feature can be overcome by using nanostructures and providing targeted delivery. Application of nanocarriers created a significant progress in gene silencing and cancer therapy, as intracellular accumulation of siRNA is improved, it is protected against degradation and its off-targeting effects are removed [66,67,68,69,70,71]. That is why experiments have been directed towards using nanoparticles for delivery of siRNAs in cancer therapy [72,73,74,75]. Polymeric nanoparticles [76], micelles [77], dendrimer [78], liposome [78], exosome [79], silicon-lipid nanoparticles [80] and cationic nanoemulsions [81] have been applied for delivery and controlled release of siRNA in cancer therapy. A recent experiment has used selenium nanostructures for delivery of Derlin1-siRNA in cervical cancer treatment. For enhancing selectivity of selenium nanoparticles toward tumor cells, its functionalization with RGDfC peptide has been performed. The RGDfC-modified selenium nanoparticles penetrate into cervical cancer cells via clathrin-mediated endocytosis and release siRNA in a sustained manner. These siRNA-loaded nanoparticles can suppress cervical cancer progression via inducing apoptosis and impairing mitochondrial function [82]. The arginine-modified calcium phosphate nanoparticles have been used for siRNA delivery and reducing expressin levels of survivin and cyclin B1 that is of importnace in sensitizing lung cancer cells to apoptosis [83]. Redox-responsive nanoparticles as smart nanocarriers have been developed for EGFR- and BRD4-siRNA delivery. Redox-sensitive nanoparticles have been modified with peptides to promote their internalization in cells and effectively suppress breast cancer progression [84]. The encapsulation efficiency of nanoparticles seems to be up to 95% [85] and they can mediate chemosensitivity via co-delivery of siRNA (KRAS) and anti-tumor drugs (gemcitabine) [86]. In the following sections, a mechanistic and in-depth discussion of using various siRNAs in PC therapy is provided. Table 1 summarizes some of the clinical trials for applications of siRNAs in cancer therapy.

## 3. SiRNA and Pancreatic Cancer Therapy

### 3.1. Proliferation and Growth

The uncontrolled growth of PC cells makes it difficult to manage this life-threatening disease. Several different factors and pathways are responsible for rapid proliferation of PC cells. A number of recent studies have focused on various oncogenic signaling networks involved in the progression of PC cells. For instance, pancreatic stellate cells are able to secrete exosomes containing miRNA-5703 to induce PI3K/Akt signaling and promote PC growth rate [87]. For example, β-catenin is another pathway whose up-regulation by Mind Bomb 1 leads to PC growth [88]. On the other hand, tumor-suppressor factors such as miRNA-573 suppress PC growth via TSPAN1 down-regulation [89]. Hence, the process of PC growth seems to be complicated and each gene can target various downstream targets to modulate PC progression [90]. The aim of current section is to show that how siRNAs can be applied in targeting pathways related to the aberrant growth of PC cells.

Precursor of nerve growth factor (proNGF) is a new and potential therapeutic target in cancer therapy. ProNGF expression undergoes up-regulation in PC and mediates metastasis of tumor cells. The lncRNA OIP5-AS1 enhances ProNGF expression via down-regulating miRNA-186-5p expression to increase PC invasion [91]. On the other hand, anoikis is a kind of apoptotic response stimulated by loss of adhesion to substrate. Reversing anoikis resistance is of importance in PC therapy [92]. Furthermore, proNGF can induce anoikis resistance [93,94]. In another study, it was found that proNGF-siRNA promotes anoikis induction in PC cells, and significantly reduces their proliferation. Following proNGF down-regulation by siRNA, autophagy inducers including autophagy-related gene 5 (ATG5) and Beclin-1 can undergo inhibition, thereby showing that apoptosis induction and autophagy inhibition can occur by proNGF-siRNA [95]. This study provides new insight about siRNA capacity in affecting interaction among programmed cell death (PCD) pathways. The apoptosis and autophagy interaction can be considered a determining factor in cancer. Inhibiting pro-survival autophagy can sensitize tumor cells to apoptosis [96,97,98]. The previous study clearly revealed that proNGF-siRNA could be beneficial in apoptosis induction via preventing autophagy [95].

NUF2 (NUF2 Component of NDC80 Kinetochore Complex) is a linker between kinetochore attachment site and tubulin subunits [99]. It has been reported that NUF2 down-regulation by RNAi leads to an impairment in attachment of kinetochore to spindle microtubules and can effectively suppress cell proliferation at prometaphase [100]. In vitro and in vivo experiments have shown that NUF2 down-regulation by siRNA leads to decreased PC cell growth. Colony formation was stopped and cell cycle arrest at G0/G1 phase occurs due to down-regulation of cyclin B1, Cdc2 and Cdc25A [101].

Receptor-associated protein 80 (RAP80) shows overexpression in PC and can mediate its progression as well as proliferation [102]. RAP80 is involved in DNA repair process via binding to BRCA1 and recruiting it to DNA damage sites [103,104,105,106,107,108]. Therefore, RAP80-siRNA can be of significant importance in reducing PC proliferation. In this case, RAP80 down-regulation by siRNA can lead to apoptosis induction via Bax up-regulation and Bcl-2 down-regulation. Caspase-8 as an executor of apoptosis was stimulated, while no changes were observed in survivin levels [109]. One of the features of cancer cells is their hypoxic microenvironment that facilitates their progression and malignancy. Hypoxia inducible factor-1α (HIF-1α) up-regulation is responsible for immunosuppression [110], radio-resistance [110], chemoresistance (gemcitabine) [111] and proliferation (Warburg effect) [112] in PC. HIF-1α-siRNA leads to a decrease in mRNA and protein levels of HIF-1α that can remarkably diminish the proliferation and induce apoptosis in PC cells [113]. Therefore, using siRNA can act as a potential strategy in suppressing PC proliferation [114]. Cell cycle arrest and apoptosis induction are major outcomes of targeting tumor-promoting factors by siRNA in PC therapy [115].

*SnoN* gene is a key member of Skt family with tumor-promoting role. This gene was first recognized due to its similarity in sequence with v-Ski and further investigation revealed that SnoN could induce growth of chicken and quail embryo fibroblasts [116,117]. The overexpression of SnoN occurs in human cancers that may result from gene amplification, transcriptional activation and enhanced protein stability [118,119,120]. In respect to the involvement of SnoN in cancer survival, its down-regulation can be considered to be a promising strategy in PC therapy. Therefore, siRNA has been introduced into PC cells for down-regulating *SnoN* gene expression. Upon *SnoN* down-regulation, PC cells undergo apoptosis, and their proliferation was interrupted [121]. It appears that anti-apoptotic proteins can be directly affected for triggering apoptosis in PC cells, instead of targeting molecular pathways that can promote PC progression. For instance, in mitochondrial pathway of apoptosis, the expression of Bcl-2 as an anti-apoptotic factor decreases. Overexpression of Bcl-2 protects cancer cells against cell death. By introducing Bcl-2-siRNA into PC cells, apoptosis can be induced [122]. The interesting point is that a variety of molecular pathways can result in increased proliferation and survival of PC cells. Nek2 is a serine-threonine kinase with potential role in both splitting centrosome and spindle formation in mammalian cells [123]. Nek2 up-regulation provides chromosome instability and aneuploidy in cancers [124]. The Nek2 inhibition appears to be advantageous in decreasing expression level of PD-L1 to enhance lymphocyte infiltration and promote anti-tumor immunity in PC suppression [125]. Hence, targeting Nek2 is of importance in suppressing cancer progression. For this reason, Nek2-siRNA has been applied for PC suppression in vitro and in vivo. Moreover, in mouse model of PC, siRNA has been introduced via a catheter. The Nek2-siRNA can impair the proliferation of PC cells and it promotes survival of xenograft mouse model. Furthermore, Nek2-siRNA can prevent liver metastasis of PC cells [126].

In addition, it has been reported that RPL1, as a ribosomal protein can be targeted in PC therapy. Moreover, down-regulation of RPL1 by siRNA leads to apoptosis and cell cycle arrest at G1 phase, and suppress DNA replication [127]. These studies advocated the fact that first step in PC therapy is identifcation of the various tumor-promoting factors. Thus, siRNAs can be designed for specific targeting of tumor cells to suppress PC proliferation and induce apoptosis [128].

Mammalian histone deacetylases (HDAC) are grouped into four different categories (I–IV) [129,130]. They participate in regulating biological processes including chromatin remodeling, gene repression, regulating cell cycle, and differentiation [129,131,132,133]. HDAC dysregulation is associated with transcription repression and inhibiting expression of tumor-suppressor genes [134,135]. HDAC1 plays a significant role in PC progression. It has been shown that HDAC1 down-regulation can lead to cancer proliferation suppression [136]. HDAC1 and HIF-1α can produce a complex in binding to hypoxia response elements (HRE) on the miR-548an promoter, down-regulating its expression and enhancing carcinogenesis in PC [137]. HDAC1 recruitment can lead to PC metastasis via reducing E-cadherin levels [138]. It has been established that HDAC1 undergoes up-regulation in PC tissues compared to normal ones. The expression of HDAC1 in PC tissues was 56.4%, while this expression was reduced significantly to 6.7% in the normal tissues. HDAC1-siRNA leads to down-regulation of this tumor-promoting factor, therefore paving the way for up-regulation of p21 and Bax in apoptosis induction in PC [139].

These studies highlighted the fact that most effective strategy for reducing proliferation rate and viability of PC cells is to affect PCD pathway, especially apoptosis activation that was discussed previously. Furthermore, autophagy is another important form of PCD that can exhibit both pro-survival and pro-death functions in cancers and inhibiting pro-survival autophagy can boost apoptosis induction in PC cells. The siRNAs have been used for down-regulating expression levels of proNGF, NUF2, RAP80, HIF-1α and SnoN to effectively impair the growth of PC cells and induce apoptosis. There are several other oncogenic pathways involved in PC progression including Wnt/β-catenin, STAT3 and NF-κB that can be focus of future studies.

### 3.2. Metastasis and Angiogenesis

The previous section clearly demonstrated that growth rate of PC cells is high and related molecular pathways can be targeted by siRNAs to effectively impair PC proliferation. Based on these previously reported studies, there are also several potential mechanisms involved in enhancing metastasis of PC cells that their targeted modulation can be advantageous in tumor treatment. The stimulation of MAPK/ERK axis by A-Raf was found to be vital in elevating migration of PC cells [140]. Furthermore, interactions occurring in tumor microenvironment can lead to PC metastasis. The recruitment of macrophages and their M2 polarization can secrete IL-6 that subsequently can induce STAT3 signaling for promoting PC migration and invasion via EMT induction [141]. STAT3 signaling can stimulate the growth and proliferation of cancer cells [142,143,144]. Both Akt and ERK1/2 molecular pathways can participate in PC metastasis and their stimulation occurs by FGF19 [145]. The metformin administration, as anti-tumor agent, substantially can reduce HNF4G expression via AMPK up-regulation to impair metastasis of PC cells [146]. Hence, PC migration is an increasing challenge and can significantly promotes aggressiveness of PC cells [147]. This section has been allocated to discuss application of siRNAs in disrupting PC invasion.

Cyclooxygenase-2 (COX-2) is an enzyme involved in the metabolic process of arachidonic acid that can actively participate in carcinogenesis [148,149,150]. COX-2 induces angiogenesis in PC through up-regulating epidermal growth factor receptor (EGFR) [151]. COX-2 overexpression demonstrates poor prognosis in PC patients [152]. COX-2 inhibitors have been applied in PC therapy due to their efficacy in angiogenesis inhibition via vascular endothelial growth factor (VEGF) down-regulation and suppressing growth [153,154]. COX-2-siRNA can trigger apoptosis and cell cycle arrest in PC cells. In tumor xenografts, COX-2-siRNA can significantly attenuate volume and weight of tumors, thus showing the efficiency of gene silencing in vivo [155].

One of the potential therapeutic targets in cancer therapy is c-Src, an important non-receptor protein tyrosine kinase. Increasing evidence demonstrates tumor-promoting role of c-Src in cancer [156,157,158]. It can promote carcinogenesis via glycolysis induction [159]. Cell adhesion molecule 1 (CADM1) as a tumor-suppressor, can down-regulate expression of c-Src in suppressing colon tumorigenesis [160]. The self-renewal capacity of breast cancer cells is also regulated by c-Src [161]. These studies highlight the role of c-Src, as a tumor-promoting factor. It seems that c-Src down-regulation by siRNA impairs progression of PC cells via inhibiting angiogenesis. The activation of angiogenesis can occur by EGFR up-regulation, a process involved in cancer metastasis and migration to the distant sites [162,163,164]. The transfection efficiency of siRNA in PC cells was more than 90% and expression of c-Src was reduced by 86.1%. Following c-Src down-regulation by siRNA, the expression of VEGF was inhibited, thus suppressing angiogenesis and cancer progression [165].

Although there have been efforts in using siRNA for reducing migration of PC cells and various factors such as COX-2 and c-Src have been affected, there is still a long way before naked siRNAs can be applied for regulating PC progression. There are several other factors such as EMT and matrix metalloproteinases (MMPs) that can also lead to metastasis of PC cells. However, there are no studies reported about using siRNAs for modulating their upstream regulators such as ZEB1/2, TGF-β and Snail, among others.

### 3.3. Immune Regulation

Transforming growth factor-β (TGF-β) is considered as a novel target in cancer therapy [166,167]. Under physiological conditions, TGF-β regulates proliferation, differentiation, survival and cell adhesion to preserve tissue homeostasis. In cancer cells, TGF-β acts as a positive factor for metastasis via EMT induction [168,169,170]. On the other hand, retinoic acid-inducible gene I (RIG-I) is affected in cancer immunotherapy by enhancing the levels of interferon and apoptosis induction [171,172,173]. A bifunctional siRNA for down-regulating TGF-β and enhancing RIG-I expression has also been designed. This improvement in function can be obtained by introducing triphosphate group at the 5′ end of siRNA. Following TGF-β down-regulation, an increase occurs in survival time of xenograft models and metastasis and invasion of PC cells undergo down-regulation. Due to RIG-I activation, immune system can be activated that can promote the levels of interferon and RIG-I and induce apoptosis [174]. In addition, TGF-β down-regulation and RIG-I up-regulation increase potential of cancer immunotherapy [175]. However, an experiment has only investigated role of siRNA in cancer immunotherapy and as immune evasion is a common phenomenon in PC [176], more studies are required to show how such genetic tools can be employed in PC treatment and activating anti-tumor immunity. Although only TGF-β signaling has been targeted in improving anti-tumor immunity in PC, there is another well-known molecular pathway, known as PD-L1/PD-1 axis that is involved in triggering immune evasion [177]. Hence, further experiments can focus on using siRNA for down-regulating PD-1 expression and promoting anti-tumor immunity against PC cells.

### 3.4. Therapy Response and Synergistic Therapy

Another potential of siRNAs lies in improving potential of chemotherapeutic agents in suppressing tumor progression [178,179]. Therefore, combination cancer therapy with siRNA-anticancer drug could be designed. For instance, L-ascorbate is not capable of preventing PC migration. Co-application of siRNA-HIF-1α and L-ascorbate resulted in ansynergistic effect in suppressing PC invasion [180]. This study demonstrated that siRNA could serve as a potential adjuvant for promoting anti-tumor activity of compounds in PC therapy. Surgery is not considered a successful option in PC therapy due to diagnosis of PC at advanced stages. Therefore, chemotherapy has been primarily used for the treatment of PC patients. However, drug resistance emerges in PC and different molecular pathways including long non-coding RNAs [181], SRPX2 [182] and USP7 [183], among others can participate in the resistance of PC cells to chemotherapy. Furthermore, drug resistant-tumor cells, especially PC cells, demonstrate high growth and invasion rates and based on previous discussions, siRNAs can be potentially beneficial in impairing tumor proliferation as well as metastasis and causing subsequent increase in chemosensitivity of PC cells.

Ribonucleotide reductase (RR) is a rate-limiting enzyme vital for DNA synthesis and replication [184]. Celastrol can suppress progression of PC cells via down-regulating RRM2 expression, showing tumor-promoting function of this pathway [185]. Exposing PC cells to RRM2-siRNA leads to an increase in apoptosis and cell growth inhibition. Noteworthy, co-administration of RRM2-siRNA and doxorubicin (DOX) can lead to synergistic effect and a four-fold increase in anti-tumor activity [186]. Gemcitabine (GEM) is another chemotherapeutic agent that its potential in PC treatment has been reduced due to emergence of drug resistance [187]. The hTERT-siRNA can increase the number of PC cells undergoing apoptosis. The hTERT down-regulation induces cell cycle arrest at G0/G1 phase and enhances number of PC cells in S and G2/M phases [188]. Following hTERT down-regulation by siRNA, expressions of Bcl-2 and COX-2, as tumor-promoting factors undergo inhibition that is of importance for inducing apoptosis in PC cells [189], and enhancing their sensitivity to chemotherapy.

Now, it is obvious that when cancer cells demonstrate malignant behavior in terms of proliferation and migration, they can obtain chemoresistance [190,191,192,193,194,195,196]. Therefore, in order to provide effective cancer chemotherapy, it is vital to suppress the various pathways that lead to cancer migration and growth [193,197]. It appears that an overexpression of heterogeneous nuclear ribonucleoprotein A2/B1 (hnRNP A2/B1) is in favor of PC growth [102]. As RNA-binding proteins, hnRNP A2/B1 participates in mRNA processing and telomere biogenesis [198]. Clinical study evaluating 42 patients with PC has shown role of hnRNP A2/B1 in PC and its association with E-cadherin, an important epithelial marker [199]. The exposure of PC cells to hnRNP A2/B1 can lead to apoptosis induction. A combination of hnRNP A2/B1 and chemotherapeutic agents such as 5-fluorouracil (5-FU), oxaliplatin and GEM can stimulate synergistic effect against PC cells. In fact, by suppressing PC growth, hnRNA A2/B1-siRNA enhances sensitivity of PC cells to chemotherapy. This combination leads to Bcl-2 down-regulation and Bax up-regulation, providing apoptotic cell death. Moreover, expression of P-glycoprotein, as a drug transporter that induces chemoresistance [200], decreases following this combination that is of importance in enhancing chemosensitivity [201].

**Figure 2:**
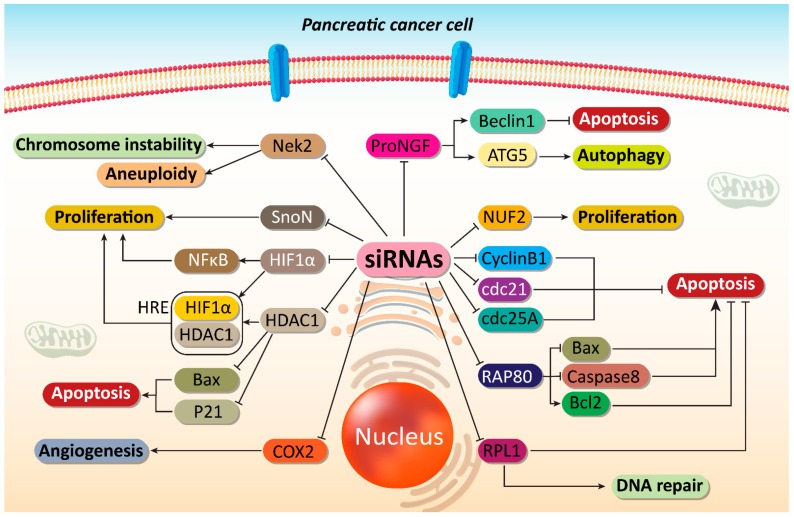
Impairing proliferation and angiogenesis of PC cells. Major molecular pathways can be targeted by siRNAs to induce apoptosis and DNA damage in PC cells. Moreover, angiogenesis responsible for PC progression can be suppressed by siRNAs in PC therapy. ProNGF, precursor of nerve growth factor; ATG, autophagy-related gene; COX2, cyclooxygenase-2; HDAC1, histone deacetylase 1; HIF-1α, hypoxia inducible factor-1α; NF-κB, nuclear factor-kappaB; siRNA, small interfering RNA; PC, prostate cancer.

**Figure 3:**
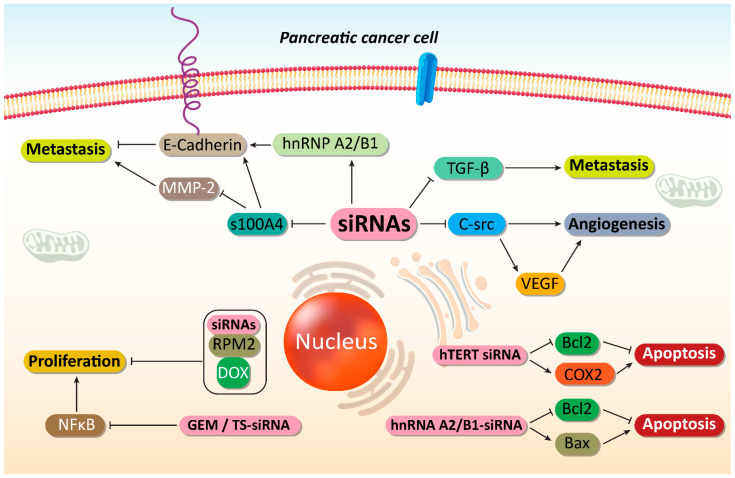
Suppressing PC metastasis and increasing their sensitivity to chemotherapy. By disrupting cancer proliferation and metastasis, as well as triggering apoptotic cell death, an increase occurs in sensitivity of PC cells to chemotherapy. SiRNAs play an important role in mediating these anticancer effects. MMp-2, matrix melloproteinase-2; hnRNP A2/B1, heterogeneous nuclear ribonucleoprotein A2/B1; TGF-β, transforming growth factor-beta; VEGF, vascular endothelial growth factor; COX-2, cyclooxygenase-2; GEM, gemcitabine; NF-κB, nuclear factor-kappaB; DOX, doxorubicin, siRNA, small interfering RNA; PC, prostate cancer.

Increasing evidence demonstrates that RR and thymidylate synthase (TS) can induce chemoresistance in cancer cells [202,203]. It has been reported that RRM2-siRNA, as a subunit of RR can promote GEM sensitivity, in vitro and in vivo [203,204]. A combination of GEM and RR- and TS-siRNA can stimulate apoptosis in PC cells and reduces their proliferation. This combination inhibits NF-κB activation following GEM administration and enhances TNF-related apoptosis-inducing ligand (TRAIL)-mediated cell death in PC cells [205]. In the previous section, it was mentioned that HIF-1α is a desirable factor for progression of PC cells. Moreover, HIF-1α down-regulation by siRNA leads to an increase in chemosensitivity of PC cells [206]. A set of tumor-promoting factors such as HIF-1α, ARNT, PFKFB4, and RBKS can be down-regulated by siRNA in inducing apoptosis and enhancing their sensitivity to chemotherapeutic agents including DOX and GEM (Figure 2 and Figure 3) [207]. The interesting point of this section is that prior studies have considered role of both molecular pathways and drug transporters in triggering drug resistance in PC and these molecular pathways and mechanisms can be markedly suppressed using siRNAs as an effective tool. Table 2 summarizes application of siRNAs in down-regulating tumor-promoting factors in PC therapy.

## 4. Chemical Modification of siRNAs

In addition to nanoscale delivery systems, stability and potential of siRNA in gene silencing can be improved by chemical modification. The RNAi activity of siRNA mainly depends on the length and structure of siRNA. The 5′-OH of siRNA possesses a phosphate group that seems to be essential for loading into RISC complex and mediating its optimal functions [213,214]. Moreover, double-stranded siRNA should have A-type duplex to provide RNAi activity [213,214,215]. The guide strand RNA-Ago2 complex has two regions including seed region and central region. The seed region has 2–8 nucleotides and is responsible for forming duplex with mRNA, while central region has 10–11 nucleotides and cleaving site of mRNA is complementary to the central region. These regions are vital for identification of target mRNA and helping RISC complex enzymes in RNA separation [216]. However, as was mentioned previously, application of siRNA as a drug has problems and chemical modification of siRNA should be performed in improving its binding affinity with mRNA, preventing enzyme degradation, providing site-specific delivery and decreasing off-targeting [217]. The sugar ring, phosphate backbones and nucleobase sites are ideal candidates for chemical modification of siRNA and improving its potential in gene expression regulation [218]. The 2′ sugar modifications of siRNA including 2′-O-methyl, 2′-fluoro (2′-F) and 2′-O-(2-methoxyethyl) (2′-MOE) are beneficial in improving biocompatibility of siRNA, decreasing its immunogenicity and providing resistance to enzyme degradation [219,220]. Furthermore, siRNA uptake by cells can be improved via conjugation of siRNAs to lipophilic agents such as ***N***-acetylglactosamine and cholesterol [221]. A new study has synthesized 2′-caged siRNA for targeting GFP gene in HEK293T cells. These 2′-caged-tethered-siRNAs were light-responsive and their activity was low in dark conditions, whereas exposing these siRNAs to light significantly elevated their RNAi activity and their potential in gene regulation [222].

It is worth mentioning that chemical modifications may negatively affect potential of siRNA in gene silencing. A recent experiment showed that KL4 peptide is optimal for increasing cellular uptake of siRNAs. Then, KL4 peptide was modified, so that hydrophobic leucine was replaced by alanine or valine. The modified peptide has cationic charge that was proper for interacting with negatively charged siRNA. However, this modification changed structure of KL4 peptide from α-helix to β-sheet that reduced potential of this peptide in enhancing siRNA efficacy [223]. Therefore, siRNA modification or changes in its conjugations should be appropriately performed to prevent unsuccessful results. For instance, conjugation of ODAGal4 to siRNA can significantly elevate the stability of siRNA and protect it against degradation by serum enzymes [224]. Another experiment highlighted the impact of chemical modification on siRNA efficacy. This study modified guide strand of siRNA by 3′ terminal modifications (2′-O-methyl versus 2′-fluoro). For siRNAs with guide strand with 20 nucleotides, such modification reduced its activity, while this is not true for guide strands with 19 or 21 nucleotides, modulating length of siRNAs can serve as determining factor and facilitate their chemical modifications. To improve efficacy of siRNAs by altering 3′ terminal 2′-O-methyl modification, another study introduced an extra 2′-fluoro modification in the seed region at guide strand position 5, but not 7 [225]. One of the primary positions that can be modified for improving potential of siRNAs to regulate gene expression is 5′ nucleobase that is responsible for the interaction between siRNA and Ago2. For instance, another study has used an adenine-derived analog, known as 6-mCEPh-pourine to modify 5′ end of siRNA that significantly improved potential of siRNA in reducing gene expression in vitro and in vivo [226]. Another experiment also revealed that 5′ end modification of siRNA by 6-mCEPh-pourine can be advantageous in promoting the generation of mature RICS by enhancing RISC stability and fixing loading orientation of siRNA duplexes [227]. Therefore, targeted chemical modifications of siRNA can be beneficial in improving its efficacy in gene silencing [228]. However, there are no prior studies reported about chemical modifications of siRNA and their possible applications in pancreatic cancer therapy. Therefore, future experiments can focus on how chemical modifications of siRNA can improve its efficacy in pancreatic cancer suppression via affecting and modulating genes as well as molecular pathways.

## 5. Co-Delivery Systems

### 5.1. Lipid-Based Nanoparticles

Although previous sections have obviously demonstrated the potential application of siRNA in PC therapy, and its efficiency in suppressing cancer proliferation and invasion, it seems that more progress can be made in effective PC therapy by loading siRNA on nanoparticles to promote its intracellular accumulation and protect against degradation [229,230,231,232,233,234,235]. In this section, different kinds of nanostructures applied for siRNA delivery in PC are discussed.

Liposomes are one of the well-known carriers for drug and gene delivery due to their great properties including long blood circulation, high stability, high drug loading and controlled release [236,237,238,239]. To enhance targeted delivery of liposomes, they can be functionalized by ligands that target overexpressed receptors on surface of cancer cells that EGFR is among them with up-regulation in PC cells [240,241]. Anti-EGFR antibodies are extensively applied in nanocarrier modification due to their low size, biocompatibility, low immunogenicity and easy conjugation on surface of nanoparticles. GE11 is a peptide that has been applied for targeting EGFR on the surface of cancer cells [242,243]. Recently, GE11 peptide antibody-targeted liposomes have been applied for co-delivery of GEM and HIF-1α-siRNA. HIF-1α up-regulation occurs in hypoxic conditions. The overexpression of HIF-1α is in favor of PC progression [244] and can mediate drug resistance [245]. Co-delivery of HIF-1α siRNA and GEM leads to a four-fold decrease in tumor growth. Apoptosis induction, DNA fragmentation and chromatin condensation can occur following administration of liposomes. It seems that using siRNA can effectively promote anti-tumor activity by two-fold [246]. This study demonstrated that how liposomes can enhance anti-tumor activity, and how siRNA can promote apoptosis induction in cancer cells by down-regulating tumor-promoting signaling pathways. Interestingly, liposomes can provide a platform for co-delivery of siRNA with other anti-tumor agents. Recently, GEM and myeloid cell leukemia 1 (Mcl-1)-siRNA have been loaded on liposomes for PC therapy (Figure 3). Liposomes demonstrated high cellular uptake, resulting in Mcl-1 down-regulation as a tumor-promoting factor for PC cells. Thus, sensitivity of PC cells toward GEM increased and an increase occurred in the number of PC cells undergoing apoptosis [247].

Overall, two major categories of nanocarriers have been applied for siRNA delivery including liposomes and polymeric nanoparticles [248,249,250,251]. The liposomes used for siRNA delivery are cationic in nature. Positively charged lipids can encapsulate a high amount of siRNA via electrostatic interaction. However, toxicity, immunostimulatory and activating inflammation are drawbacks of cationic liposomes [249,252]. Similarly, polymers applied for siRNA delivery possess positive charge and severe toxicity is still a major challenge for these kinds of nanostructures. To promote efficiency of polymeric nanoparticles in siRNA delivery, they are designed with high charge densities that can be modulated based on their toxicities.

It has also been reported that lipid-polymer hybrid nanoparticles are potential carriers in siRNA delivery. The structure of lipid-polymer hybrid nanoparticles includes a polymeric core surrounded by a single layer or bilayer lipid shell, combining benefits of both liposomes and polymeric nanoparticles. The cationic core is responsible for encapsulation of siRNA, and can provide protection, biocompatibility as well as in vivo stability [253,254]. Lipid-polymer hybrid nanoparticles have been designed for co-delivery of GEM and HIF-1α-siRNA in PC therapy. The negatively charged HIF-1α-siRNA is encapsulated on the surface, while GEM is embedded into hydrophilic core. Then, they are encapsulated by PEGylated lipid bilayer that can prevent siRNA degradation and aggregation as well as GEM leakage. These nanocarriers can enhance lifetime in bloodstream and increase tyhe drug release via penetrating into tumor vasculature. In vivo and in vitro experiments demonstrated down-regulation of HIF-1α and effective delivery of GEM in suppressing PC progression [255].

The different kinds of nanocarriers applied for siRNA delivery not only prevent siRNA degradation, but also inhibit renal clearance following systemic administration [42]. Moreover, nanostructures possess a size lower than 100 nm that is of importance in accumulating in tumor site via defective neovasculature surrounding tumor, known as enhanced permeability and retention (EPR) effect [256,257]. PEG-based block catiomers can spontaneously assemble siRNA into micelles, providing a biocompatible PEG shell for siRNA delivery [258]. Chemical cross-linking and hydrophobic interactions derived from hydrophobic moieties can enhance stability of micelles [42,259]. Furthermore, targeted delivery of micelles can be improved via using ligands specifically targeting overexpressed receptors on surface of cancer cells [260]. For this reason, Min and colleagues have designed antibody fragment (Fab’)-installed polyion complex (PIC) micelles for enhancing siRNA delivery in PC cells. The prepared micelles demonstrated diameter as low as 40 nm. PIC micelles demonstrated high affinity to PC cells overexpressing tissue factor (TF). This led to high internalization and penetration into PC cells. Subsequently, highest decrease occurred in expression of polo-like kinase 1 mRNA, after using PIC micelles containing siRNA [261].

### 5.2. Polymeric Nanoparticles

Polymeric nanoparticles are considered to be ideal candidates in siRNA delivery because of their biodegradability [238,239,262]. Biodegradable charged polyester-based vectors (BCPVs) can be degraded under the physiological conditions and have been successfully applied for siRNA delivery [263]. On the other hand, *K-Ras* family proteins are guanine nucleotide binding proteins capable of regulating activity of pancreatic cells and are involved in modulating proliferation, apoptosis and migration [264,265,266]. Following *K-Ras* mutation, several downstream signaling networks were activated that imparted cancer malignancy [267].

The role of *K-Ras* in PC growth was confirmed when the cancer cells were exposed to *K-Ras*-siRNA and the number of PC cells undergoing apoptosis demonstrated an increase [212]. *K-Ras*-siRNA-loaded BCPVs have been designed for PC therapy. After 72 h, BCVPs demonstrate good accumulation in cancer cells and they can diminish mRNA and protein expression up to 50%. These siRNA-loaded nanostructures effectively penetrate into PC cells to induce apoptosis, and growth inhibition [268]. Efficacy of polymeric nanocarriers in siRNA delivery can be boosted via their surface modifications. The internalizing RGD peptide (iRGD) functions by binding to integrins overexpressed on tumor vasculature [269]. Using iRGD also significantly elevated tumor-penetrating capability [270,271]. Surface modification by iRGD is associated with an increase in siRNA delivery to cancer cells, and subsequent enhance in gene silencing and reducing tumor growth [272]. In fact, RGD can bind to αvβ3/5 integrins on surface of cancer cells, leading to an increased transfection efficiency [273]. One of the achievements of using polymeric nanoparticles for siRNA in PC therapy is EPR effect. However, in PC cells, due to hypovascularity and dense desmoplastic stroma, intravenous administration (IV) can decrease efficiency of nanostructures in siRNA delivery and cancer elimination, Hence, intraperitoneal administration has been suggested [274].

It has been established that hypoxia is in favor of cancer growth by activation of HIF-1α signaling and its downstream targets [275]. Interestingly, it was reported that endothelial PAS domain protein 1 (EPAS1) is also activated under hypoxic conditions. EPAS1 overexpression is an obvious finding in cancer and can enhance cancer metastasis via EMT induction [276,277]. The polyethylenimine-poly(lactide-coglycolide) (PLGA)/poloxamer nanoparticles loaded with EPAS1 siRNA have been applied for PC therapy. In vitro and in vivo experiments demonstrated that EPAS1 down-regulation in PC cells due to targeted delivery, resulted in a reduction in cancer proliferation and the number of microvessels [278]. G protein-coupled receptor (GPCR) 87 located on chromosome 3q24 is involved in encoding protein that has an extracellular N terminus, seven helices, three intracellular loops, three extracellular loops and an intracellular C terminus [279,280]. GPCRs are located on the surface of cells and their overexpression can lead to the cancer survival [281,282].

The up-regulation of GRP78 occurs in PC that can mediate chemoresistance via inducing activity of ATP-binding cassette (ABC) drug transporters [283]. GRP78 can induce Yes-associated protein (YAP) in providing radio-resistance [284]. Recently, PLGA nanoparticles containing GRP78-siRNA have been prepared for PC therapy. To prevent polydispersity and large size of nanoparticles, mild agitation was used for encapsulating siRNA in PLGA nanoparticles. The synthesized PLGA nanoparticles demonstrate low size of 92 nm. The expression of GRP8 decreased up to 83.9% that showed high cytotoxicity of these nanocarriers against PC cells [285]. The advantages of using nanoparticles is that the capability of siRNAs in gene silencing increases due to enhanced intracellular accumulation. That is why experiments have focused on using nanostructures for siRNA delivery in cancer therapy [286]. Local drug EluteR (LODER) is a biodegradable polymeric matrix applied for siRNA in PC therapy. It can protect siRNA against degradation and has high biocompatibility, appropriate for siRNA delivery [287]. Moreover, polymeric nanoparticles can provide endosomal escape of siRNA that is of importance in enhancing its efficacy in gene silencing and suppressing PC progression [288]. An experiment has revealed that using polymeric nanoparticles can provide transfection efficiency as much as 90% [289]. It appears that these two factors are important in designing smart siRNA polymeric carriers including low pKa amines and hydrophobic moieties inside chain [290].

### 5.3. Carbon-Based Nanoparticles

Two-dimensional (2D) nanomaterials are extensively applied in the field of medicine due to their great electronic, optical and chemical characteristics [291,292]. Graphene is a well-known 2D nanomaterial capable of gene delivery in cancer [293]. To improve biocompatibility of graphene nanomaterials, their surface can be functionalized by compatible polymers such as polyethylene glycol (PEG) [294]. The PEGylated graphene oxide nanosheets have been designed for delivery of siRNA in PC therapy. These biocompatible nanomaterials can successfully down-regulate HDAC1 and K-Ras in suppressing proliferation and triggering apoptosis and cell cycle arrest. In vivo experiment demonstrated an inhibition of cancer growth as much as 80% following delivery of siRNA by graphene nanosheets [295].

### 5.4. Dendrimers

As it was mentioned, naked siRNAs can poorly penetrate into the cells due to their high molecular weights and high density of negative charge [248]. Moreover, nucleases can easily degrade siRNA in plasma, thus remarkably reducing its efficiency in gene silencing [278]. Dendrimers are a class of materials applied for gene and drug delivery due to their highly branched and precise molecular structures [296,297]. Dendrimers can provide endosomal release and intracellular uptake of siRNA in cancer cells [298]. Furthermore, dendrimers can be applied for co-delivery of chemotherapeutic agents and siRNA in effective cancer chemotherapy by down-regulating the tumor-promoting molecular pathways and enhancing cytotoxicity of anti-tumor agents [299]. On the other hand, targeted delivery of dendrimers can be improved using plectin-1 targeted peptide (PTP), as a biomarker of PC. PTP peptide modified dendrimers have been designed for co-delivery of paclitaxel (PTX) and TR3-siRNA in PC therapy. These smart nanostructures are redox-responsive and can induce endosomal escape and provide siRNA against degradation. Enhanced intracellular accumulation of TR3-siRNA and PTX resulted in an increase in their cytotoxicity against PC (in vitro and in vivo) [300].

### 5.5. Metal-Based Nanoparticles

Superparamagnetic iron oxide nanoparticles (SPIONs) are a novel kind of nanocarriers being used in field of cancer therapy. They have been used for gene and drug delivery in cancer eradication. SPIONs can enhance intracellular accumulation of DOX in breast cancer cells to promote apoptosis induction [301]. They can also be considered to be acting as radiosensitizer in cancer therapy [302]. SPIONs are biocompatible with low size. They can carry siRNA in down-regulating oncogene signaling pathways (HIF-1α/CD73) in disrupting cancer progression [303]. On the other hand, polo-like kinase 1 (PLK1) is a tumor-promoting factor and its overexpression is correlated with an increase in PC growth and induction of chemoresistance [304,305]. Tumor-suppressor factors such as miRNA-23a diminish PLK1 expression in inhibiting PC proliferation and invasion [306]. In an experiment, SPION have been designed for delivery of PLK1-siRNA in PC therapy. To promote specificity of SPIONs in targeting PC cells, their surface has been modified with a tumor-selective peptide (EPPT1). Remarkable accumulation of SPIONs carrying siRNA occurs in cancer cells, thus causing PLK1 down-regulation. This can induce apoptosis and suppress proliferation and growth of PC cells. The interesting point is that in vivo and in vitro experiments have confirmed the potential role of siRNA-loaded SPIONs in PC therapy [307]. Clinical studies will shed more light on efficacy of SPIONs in gene delivery for cancer therapy.

Gold nanostructures are promising candidates for nucleic acid delivery due to their adjustable size, multiple functional capabilities and surface properties [308,309,310,311,312,313]. Gold nanoparticles can be applied for nucleic acid delivery with minimum toxicity and off-targeting [314,315,316]. On the other hand, nerve growth factor (NGF) is considered to be an inducer of cancer proliferation and its inhibition can be correlated with apoptosis induction [317]. NGF can promote proliferation of PC cells via inducing phosphoinositide 3-kinase (PI3K)/protein kinase B (Akt) axis [318]. Gold nanoclusters have been designed for delivery of NGF-siRNA in PC therapy. Moreover, using gold nanoparticles for delivery both stability and intracellular accumulation of siRNAs can be increased effectively. NGF-siRNA-loaded gold nanoparticles can markedly suppress cancer growth by causing NGF down-regulation without exhibiting any major side effects [319].

Mesoporous silica nanoparticles (MSNPs) are considered to be efficient drug delivery platforms due to their characteristics including that of large surface area and ordered porous channels [320,321]. In addition, MSNPs have been found to be safe, biocompatible and biodegradable [322,323]. Recently, much attention has been directed towards using MSNPs for siRNA delivery in cancer therapy. MSPNs can be administered through intravenous route in mouse models of breast cancer, while their potential in siRNA delivery is maintained [324]. MSNPs can provide a platform for co-delivery of siRNA with anti-tumor agents [325]. Their efficacy in siRNA delivery can be improved via surface modification by chloroquine [326]. An experiment has designed MSNP for siRNA delivery in PC therapy. To enhance intracellular uptake of MSNPs, their surface has been coated with polyethyleneimine (PEI). In addition to increasing cell internalization, PEI can provide a cationic charge that is in favor of encapsulating negatively charged siRNA. The drawback of PEI is negatively affecting biocompatibility profile of MSNPs, but these effects have been found to be only partial. Moreover, to reduce the cytotoxicity against normal cells, 10 kD PEI can be used instead of 25 kD PEI. They can effectively deliver siRNA and PTX to PC cells, as was shown by fluorescence technique (70% transfection efficiency) [327].

### 5.6. Viral Vectors

**Figure 4:**
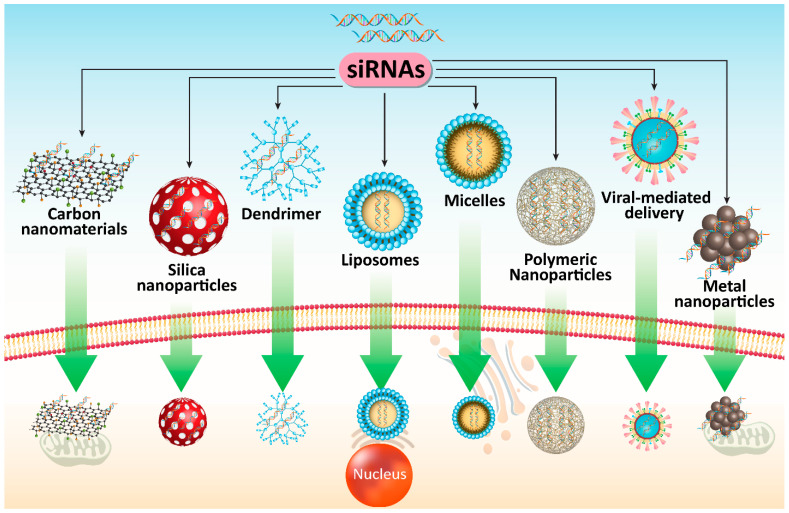
Different co-delivery systems for siRNA in PC therapy. Enhancing intracellular accumulation, protecting against degradation and increasing efficacy for gene silencing can be obtained using viral and non-viral vectors. SiRNA, small interfering RNA; PC, prostate cancer.

**Figure 5:**
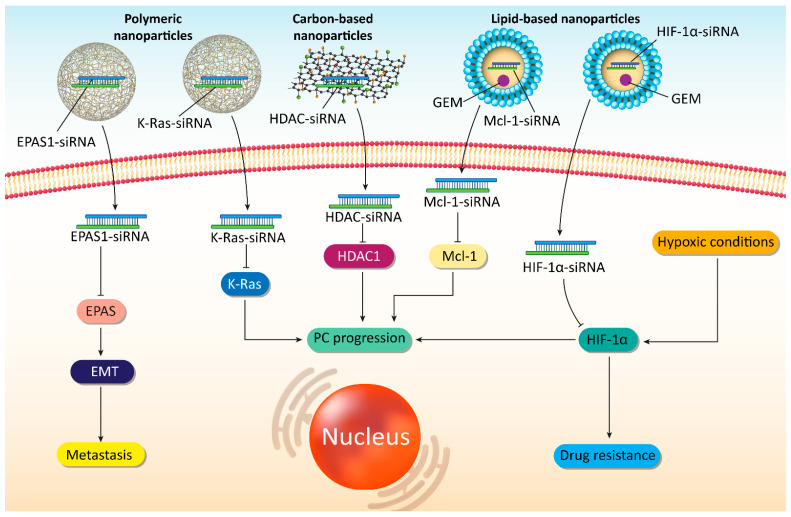
The potential of siRNA-loaded nanoparticles in affecting molecular pathways in PC therapy.

Although previous sections were allocated to application of nanomaterials as non-viral vectors for siRNA delivery in cancer therapy, it seems that viral vectors are also capable of effective delivery of siRNA to PC cells that has been discussed in this section. Increasing evidence demonstrates potential of viral vectors for siRNA delivery in cancer therapy [328,329,330]. Retroviral vectors are applied for siRNA generation induced by either U6- or H1-RNA promoter to provide stable knock-down of targeted gene [328,329]. However, retroviral vectors have a narrower spectrum of cell types compared to adenovirus due to integrating into genome [331,332]. Recombinant adenoviruses are considered to be ideal candidates for cancer gene therapy. For generating recombinant adenoviruses, a simple approach known as AdEssay has been used [333]. Recombinant adenoviruses can be applied for inducing persistent loss of functional phenotypes. In PC therapy, *K-Ras*-siRNA can be delivered by adenoviruses in silencing its expression and suppressing PC progression [334]. Another study has used adenovirus vector for delivery of Mcl-1 in PC therapy. Mcl-1 can regulate mitochondrial activation and undergoes up-regulation in several cancers [335,336,337]. Following introduction of Mcl-1-siRNA-loding adenovirus, a significant decrease occurs in PC cells that can induce apoptosis via caspase-3 up-regulation. It seems that Mcl-1-down-regulation by adenovirus is in favor of promoting radiosensitivity of PC cells [338]. These studies demonstrated efficiency of adenoviruses in down-regulating tumor-promoting factors such as Gli1 in inhibiting PC proliferation and viability [339]. Figure 4 and Table 3 describe the role of different co-delivery systems for application of siRNAs in PC therapy. Figure 5 illustrates the potential of siRNA-loaded nanoparticles in affecting different molecular pathways in PC therapy.

## 6. SiRNA and Pancreatic Cancer: Clinical Applications

With respect to the various challenges associated with PC treatment in clinical courses, introducing novel therapeutics is of great importance. Based on the previous sections, siRNAs have shown high potential in PC treatment by targeting tumor-promoting molecular pathways and sensitizing these malignant cells to death. A search on clinicaltrials.gov demonstrates that clinical studies are currently ongoing about potential applications of siRNAs in PC therapy. The mutation in KrasG12D is responsible for PC progression and spread into various regions of the body. A phase I clinical trial by MD Anderson Cancer Center is going on to evaluate role of exosomes derived from mesenchymal stem cells for delivery of KrasG12D-siRNA in PC treatment. This clinical experiment aims at determining the optimal dose and adverse effects of exosomes containing KrasdG12D-siRNA in PC treatment (NCT03608631). When PC cells undergo metastasis and spread in body, it is impossible to treat PC with surgery or tumor resection. Therefore, use of novel strategies has been recommended and siRNAs can also be among them. Immune evasion is a common phenomenon in PC and there are several factors found in immune cells that can hamper their capacity in PC cell eradication. A clinical experiment performed by Wake Forest University Health Sciences aims to develop siRNA-transfected peripheral blood mononuclear cells APN401 in treatment of PC patients (NCT02166255). The major aim of these clinical trials is to show safety profile and toleration of siRNA in PC patients (NCT01188785). One of the limitations of these clinical trials is the limited number of participants. For instance, there clinical trials have been performed for using siRNA and the numbers of participants are 44 (NCT03819387), 29 (NCT01808638) and 24 (NCT00689065). Noteworthy, a phase II clinical trial has been recruited and is going to evaluate siRNA-G12D LODER in combination with gemcitabine and nab-paclitaxel in treatment of PC patients. This clinical trial has 80 participants that is slightly better as compared to the previous ones (NCT01676259). However, patient population is still limited and additional clinical studies are needed with greater number of PC patients. Furthermore, there are no clinical trials reported that have used siRNA-loaded nanoparticles for treatment of PC patients and these can also be the focus of future studies.

## 7. Conclusions and Remarks

In the current review, different aspects of using siRNAs and their delivery systems in PC therapy were discussed. Activation of tumor-promoting factors has been found to responsible for increase in proliferation and metastasis of PC cells. To suppress PC growth and viability, siRNAs have been developed for down-regulating the various tumor-promoting factors including proNGF, RAP80, NUF2, SnoN, HIF-1α, COX-2 and Nek2. Following down-regulation of aforementioned factors, a significant decrease occurs in PC progression, thus showing potential benefits of application of siRNAs in PC therapy. Noteworthy, a variety of factors are also involved in PC migration and invasion. Thus, siRNAs have been designed for inhibiting tumor-promoting factors including TGF-β, c-Src, and HIF-1α to disrupt PC progression and metastasis. The interesting point is that when an increase in the proliferation and migration of PC occurs, they can also obtain resistance to chemotherapy and radiotherapy, which can also be targeted using specific siRNAs.

Despite achieving promising results following siRNA application, it appears that more advancement can be made in suppressing PC progression using optimal delivery systems to prevent siRNA degradation, inhibit off-targeting and enhance targeted delivery. To date, a wide variety of nanocarriers including carbon nanomaterials, micelles, liposomes, polymeric nanoparticles, metal nanoparticles, dendrimers and silica nanoparticles have been developed for enhancing delivery of siRNAs for PC therapy. Using these nanoarchitectures can significantly enhance efficacy of siRNA in gene silencing and promote their intracellular accumulation. It is worth mentioning that viral vectors have also been developed for siRNA delivery in PC therapy. Overall, the studies conducted so far have revealed that siRNAs along with their associated delivery systems can serve as powerful tools in PC therapy. Hence, future studies can focus on developing novel carriers for targeting the various tumor-promoting factors in PC therapy.

## Figures and Tables

**Table 1:** Clinical trials using siRNAs in the treatment of cancer patients.

siRNA	Cnacer Type	Phase	Aim	Trial Number
Atu027	Advanced solid tumors	Phase I	34 participants that receive eight treatments within 4 weeks The aim is to determine best and optimal dose in cancer treatment	NCT00938574
EphA2	Advanced or recurrent solid tumors	Phase I	The EphA2 leads to cancer growth and progression siRNA targeting EphA2 paves way in cancer treatment Determing dose and side effects	NCT01591356
CALAA-01	Solid tumors	Phase I	Determing pharmacokinetics and safety profile	NCT00689065
PLK1	Liver cancer	Phase I	Testing a new drug, known as TKM-080301 that is a liposomal nanoformulation containing siRNA-PLK1 in cancer treatment	NCT01437007
siRNA taregting immunoproteasome	Melanoma	Phase I	The aim is to improve anti-tumor immunity and prevent immune evasion of cancer cells	NCT00672542
MYC	Solid tumors Lymphoma Multiple myeloma	Phase I	Encapsulation of siRNA-MYC by lipid nanoparticlesin cancer therapy	NCT02110563
PD-L1/PD-L2	Heamtological malignancies	Phase I Phase II	Developing a new vaccine and mediating immunotherapy	NCT02528682

**Table 2:** Interference with PC progression via application of siRNAs.

In Vitro/In Vivo	Cell Line/Animal Model	SiRNA	Outcomes	Refs
In vitro In vivo	PANC-1 and Sw1990 cell lines Xenograft nude mice	NUF2	Cell cycle arrest at G0/G1 phase Down-regulation of Cdc2, Cyclin B1 and Cdc25A Suppressing carcinogenesis	[101]
In vitro	AsPC-1, SUIT-2, and Panc-1 cells	Survivin	Reducing promoter activity and mRNA expression of survivin Inducing caspase-3 expression and DNA fragmentation Enhancing radiosensitivity	[208]
In vitro	SW1990 and Capan-2 cells	RAP80	Down-regulating Bcl-2 and up-regulating Bax Inducing apoptotic cell death Increasing TRAIL-mediated apoptosis Promoting GEM sensitivity	[109]
In vitro	Panc-1 and BxPC3 cells	Survivin	Decreasing mRNA and protein levels of survivin Suppressing cell proliferation Triggering cell cycle arrest at G0/G1 phase	[209]
In vitro	BxPC3 cells	S100A4	Reducing gene expression by 17% Down-regulating MMP-2 Enhancing levels of E-cadherin and TSP-1 Suppressing cancer invasion and metastasis	[210]
In vitro	PANC-1, MIA-PaCa-2 and ASPC-1 cells	TrKA	TrKA down-regulation is associated with GEM sensitivity Inducing apoptotic cell death Inhibiting PI3K/Akt signaling pathway	[211]
In vitro	BxPC3 cells	hTERT	Apoptosis stimulation Cell cycle arrest at G0/G1 phase Enhancing GEM sensitivity	[188]
In vitro	MiaPaCa2 cells	HIF-1α	Interfering with cancer proliferation Apoptosis induction Disrupting cancer growth under hypoxic conditions	[113]
In vitro	PaTu8988 cells	DNMT1	Apoptosis induction and inhibiting tumor growth by cell cycle arrest (S phase) DNMT1 down-regulation and subsequent activation of hMLH1 as a tumor-suppressor factor	[115]
In vitro	PANC-1 cells	RRM2	Exerting synergistic effect with doxorubicin and enhancing cytotoxicity against cancer cells by 4-fold	[186]
In vitro	MiaPaCa-2 cells	*K-Ras*	Down-regulating *K-Ras* expression Triggering apoptosis	[212]
In vitro	SW1990 cells	SnoN	Down-regulating SnoN expression and reducing cancer cell proliferation Apoptosis induction	[121]
In vitro	SW1990 and BxPC-3 cells	hnRNP A2/B1	Stimulating apoptosis via Bcl-2 down-regulation and Bax up-regulation Mediating TRAIL-induced apoptosis P-glycoprotein down-regulation Suppressing cancer metastasis via enhancing E-cadherin levels	[201]
In vitro In vivo	Capan-2 cells Nude mice	COX-2	Cell cycle arrestApoptosis induction Decreasing cancer cell proliferation	[155]
In vitro	PaTu8988 cells	HDAC-1	Disrupting cancer growth and survival Inducing cell cycle arrest (S phase) and apoptosis Enhancing Bax and p21 expressions	[139]
In vitro In vivo	PANC-1 and BxPC-3 cells	RPL21	Cell cycle arrest at G1 phase Apoptosis induction via mitochondrial pathway Caspase-8 activation	[127]

**Table 3:** Co-delivery systems for siRNA in PC therapy.

Vehicle	SiRNA	In Vitro/In Vivo	Cell Line	Surface Modification	Particle Size (nm) Zeta Potential (mV) Encapsulation Efficiency (%)	Remarks	Refs
Polymeric nanoparticles	*K-Ras*	In vitro In vivo	KPC-derived cell lines and MIA PaCa-2 cells	RGD	Not reported	Gene down-regulation efficiency more than 95% High cellular uptake Great internalization Suppressing PC progression	[272]
Polymer hybrid nanoparticles	VEGF	In vitro In vivo	BxPC3 cells	N/A	120–140 nm 35 mV	100 nm in size, spherical shape and narrow dispersion High gene silencing efficiency Reducing tumor growth	[290]
Lipid-polymer hybrid nanoparticles	HIF-1α	In vitro In vivo	PANC-1 cells	N/A	120–140 nm −34 mV	Co-delivery of GEM and siRNA in exerting synergistic effect Prolonged lifetime in bloodstream and improved drug release via the enhanced tumor vasculature effect in tumor tissues Suppressing tumor growth and metastasis Down-regulating HIF-1α Enhancing GEM sensitivity	[255]
Polymeric nanoparticles	KRAS	In vivo	KPC8060 cells	N/A	Not reported	Intraperitoneal injection enhances intracellular accumulation of nanoparticles to intravenous administration (15-fold higher) Enhancing infiltration of T cytotoxic cells Inducing delay in tumor growth Suppressing metastasis Increasing survival	[274]
Polymeric nanoparticles	EPAS1	In vitro In vivo	BxPC3 cells	N/A	160–220 nm −0.41 mV 40%	Prolonged-release behavior Suppressing cancer growthTriggering apoptotic cell death Down-regulating EPAS1 Reducing tumor vessels and VEGF inhibition	[278]
Polymeric nanoparticles	GPR87	In vitro	HEK293T cells	N/A	Average size of 100–200 nm Up to −15 mV Up to 31.14%	Reducing gene expression up to 87% High efficiency and cytotoxicity against cancer growth	[285]
Polymeric nanoparticles	K-Ras	In vivo	MiaPaCa-2 cells	N/A	97.99 nm 39.71 mV	High biocompatibility Potentiality in siRNA delivery and gene silencing in suppressing cancer progression	[268]
Polymeric nanoparticles	K-Ras	In vitro In vivo	PANC-1 and BxPC3 cells	N/A	Not reported	Apoptosis stimulationCell cycle arrest at G0/G1 phase Enhanced efficiency in gene silencing	[286]
Gold nanocluster	NGF	In vitro In vivo	Panc-1 cells Tumor models	N/A	Not reported	High cellular uptake and intracellular accumulation NGF down-regulation Inhibiting PC proliferation and viability	[319]
Liposome	HIF-1α	In vitro In vivo	Panc-1 cells	GE11	166.4 nm 22.5 mV	Enhancing GEM sensitivity of cancer cells via HIF-1αdown-regulation	[246]
Liposome	Mcl-1	In vitro	PANC-1 and BxPC3 cells	N/A	N/A	Increased efficiency in down-regulating Mcl-1 Suppressing GEM resistance	[247]
Peptide nanoparticles	KRAS	In vitro In vivo	KPC-1 murine PDAC cells	N/A	Not reported	Precision delivery to tumor site High cellular uptake Potentiality in gene silencing	[229]
Single wall carbon nanotubes	K-Ras	In vitro	PANC-1 cells	N/A	110–150 nm +40 mV	High transfection efficiency and cellular internalization Down-regulation of target gene	[340]
Graphene oxide nanosheet	HDAC1 K-Ras	In vitro In vivo	MIA PaCa-2 cells	N/A	550–637 nm +32 to +29 mV	Synergistic effect by combining two siRNAs Simultaneous phototherapy and gene therapy Apoptosis induction Cell cycle arrest and inhibiting cancer growth Reducing tumor growth by more than 80%	[295]

## Abbreviations

PC	Pancreatic cancer
siRNA	Small interfering RNA
BBB	Blood-brain barrier
BTB	Blood-tumor barrier
RNAi	RNA interference
mRNA	Messenger RNA
RISC	RNA-induced silencing
Ago	Argonaute
HSP27	Heat shock protein 27
PARP	Poly (ADP-ribose) polymerase
ESM-1	Endothelial cell-specific molecule-1
proNGF	Precursor of nerve growth factor
ATG5	Autophagy-related gene 5
RAP80	Receptor-associated protein 80
HIF-1α	Hypoxia inducible factor-1α
NF-κB	Nuclear factor-kappaB
COX-2	Cyclooxygenase-2
EGFR	Epidermal growth factor receptor
VEGF	Vascular endothelial growth factor
HDAC	Histone deacetylase
HRE	Hypoxia response element
HPA	Heparinase
FGF2	Fibroblast growth factor 2
SDC1	Syndecan-1
EMT	Epithelial-to-mesenchymal transition
NELFE	Negative elongation factor E
NDRG2	N-Myc downstream-regulated gene 2
lncRNA	long non-coding RNA
miRNA	microRNA
MMP	Matrix metalloproteinase
TGF-β	Transforming growth factor-beta
RIG-I	Retinoic acid-inducible gene I
CADM1	Cell adhesion molecule 1
RR	Ribonucleotide reductase
DOX	Doxorubicin
GEM	Gemcitabine
hTERT	Human telomerase reverse transcriptase
hnRNP	Heterogenous nuclear ribonucleotide protein A2/B1
TS	Thymidylate synthase
TRAIL	TNF-related apoptosis-inducing ligand
Mcl-1	Myeloid cell leukemia 1
EPR	Enhanced permeability and retention
Fab	Fragment
PIC	Polyion complex
TF	Tissue factor
BCPVs	Biodegradable charged polyester-based vectors
iRGD	Internalizing RGD peptide
IV	Intravenous
EPAS1	Endothelial PAS domain protein 1
PLGA	Polyethylenimine-poly (lactic-coglycolide)
GPCR	G protein-coupled receptor
ABC	ATP-binding cassette
YAP	Yes-associated protein
LODER	Local drug EluteR
2D	Two-dimensional
PEG	Polyethylene glycol
PTX	Paclitaxel
PTP	Plectin-1 targeted peptide
SPIONs	Superparamagnetic iron oxide nanoparticles
PLK1	Polo-like kinase 1
NGF	Nerve growth factor
PI3K	Phosphoinositide 3-kinase
Akt	Protein kinase-B
MSNPs	Mesoporous silica nanoparticles
PEI	Polyethyleneimine
